# Multidimensional Scales of Perceived Self-Efficacy (MSPSE): Measurement invariance across Italian and Colombian adolescents

**DOI:** 10.1371/journal.pone.0227756

**Published:** 2020-01-15

**Authors:** Emanuele Basili, Maryluz Gomez Plata, Carmelina Paba Barbosa, Maria Gerbino, Eriona Thartori, Carolina Lunetti, Liliana Maria Uribe Tirado, Marcela Ruiz García, Bernadette Paula Luengo Kanacri, Gonzalo Tamayo Giraldo, Mariela Narvaez Marin, Fiorenzo Laghi, Concetta Pastorelli

**Affiliations:** 1 Department of Psychology, Sapienza University of Rome, Rome, Italy; 2 Department of Psychology of Development and Socialization Processes, Sapienza University of Rome, Rome, Italy; 3 Grupo de investigación Cognición y Educación, Programa de Psicologia, Universidad del Magdalena, Santa Marta, Colombia; 4 Facultad de Psicología, Universidad de San Buenaventura, Medellin, Colombia; 5 Escuela de Psicología, Pontificia Universidad Católica de Chile, Santiago, Chile; 6 Facultad de Ciencias Sociales y Humanas, Universidad de Manizales, Manizales, Caldas, Colombia; University of Rome, ITALY

## Abstract

Multidimensional Perceived Self-Efficacy Scale for Children has been developed as an important tool to measure Self-Efficacy in school contexts. The present study assesses the measurement invariance of the MSPSE across two samples of Italian and Colombian adolescents using Multi-sample Confirmatory Factor Analysis. Participants were Italian (N = 564) and Colombian (N = 645) students attending the 7^th^ grade (age 12–13) drawn from a residential community near Rome and three Colombian cities: Medellin, Manizales and Santa Marta. Findings from gender invariance provide high support for full and partial invariance among Colombian and Italian adolescents respectively. Cross-national comparison showed partial scalar invariance between Italy and Colombia, with Italian students perceiving themselves as more efficacious on Academic, Social and Self-Regulatory dimensions. MSPSE’s structural validity has been confirmed, along with its three-factor-structure across gender, for the Italian and Colombian samples. The findings support the invariance and the validity of this scale to measure Self-Efficacy in school contexts from a cross-cultural perspective.

## Introduction

According to Social Cognitive Theory, Perceived Self-efficacy is a key mechanism in the exercise of human agency within a causal structure involving triadic reciprocal causation between the person, the environment, and behavior [1]. Self-efficacy beliefs are the convictions individuals hold about their capacity to reach desired outcomes, to overcome impediments, to resist adversities, to self-regulate in the face of pressing circumstances, to discern among many competing alternatives, and to negotiate important life transitions [2].

The appraisal of children’s efficacy beliefs derives from three relevant contexts in children’s life: the family, peers and the school. The family is the first source of efficacy information for children. During the early period of life, children are completely dependent, and parents mediate their transactions with the environment. Responsive parents create the opportunity for their children to learn better communication, social and cognitive skills [3]. They expose their children to experiences of success which contribute to the build-up of an appraisal of their efficacy.

Peers are the second source of efficacy [4]. As the child rapidly expands social relationships, peers become an important source of information concerning one’s capabilities. The experience of new relationships and observing their peers and confronting with them represent a valuable source of information about their capabilities.

School is the third source of efficacy information [5]. During a child’s formative period, educators contribute to the formation of a child’s intellectual efficacy. A good part of children’s appraisals of their efficacy derive by the way teachers evaluate their performances and help them to develop self-regulatory skills in managing learning activities [6]. Children become active contributors to their own development through their exercising of personal agency in different phases of development. In our study we focus on the transition to adolescence as a period of life in which many developmental changes occur at biological, personal and social level. As a consequence, successful adaptation requires the adolescents to exert an expanding agentic role in multiple scholastic, familial, and social domains of their life. Here, we focused on a differentiated set of perceived self-efficacy in two relevant spheres of adolescent’s life: academic and social (peers).

Academic self-efficacy is a crucial form of self-efficacy during adolescent’s development and adjustment [7, 8]. Previous studies conducted in the academic context have shown perceived self-efficacy beliefs to be important contributors to students’ academic achievements and personal success. In particular, academic self-efficacy beliefs affect perceptions of capabilities in the scholastic subjects, as well as, in self-regulatory processes conducive to learning [9, 10]. Students with higher self-efficacy beliefs are better able to manage their own learning, more likely to do better academically, to use their own performances as a guide for assessing their self-efficacy and finally to successfully complete their education [2, 11–13]. Academic self-efficacy beliefs also influence career paths and job attainment, as higher levels of self-efficacy influence individuals to consider a wider range of career options [9]. In the longitudinal study of Caprara et al. [14], it has been found a decline of *self-regulatory learning* efficacy–as a specific facet of Academic Efficacy-from junior to senior high school, and results evidenced that especially those students who experienced the lowest decline in self-regulatory efficacy reached higher grades and had a greater chance of remaining in school.

In the social context, perceived self-efficacy beliefs exert a crucial influence on adolescents’ capabilities to successfully interact with their own peers, namely *Social efficacy* [15], and to self-regulate their own behavior in transgressive contexts, namely, *Self-regulatory efficacy*. Previous studies evidenced that high levels of *Social efficacy* beliefs foster adolescents’ positive social relationships through their capacity to deal properly with the daily relationships in the classroom and mitigate the negative consequences of unsuccessful social interactions, such as those characterized by aggressive and submissive behaviors, or those related to ignoring or denying others’ points of view [2]. *Social Self-Efficacy* has been consistently linked to better psychological and behavioral adjustment, for example lower levels of depression [9, 16, 17], other internalizing symptoms [18], and delinquent behavior [16, 19]. In addition, students’ capacity to resist peer pressure, that is Self-regulatory efficacy, plays a relevant role when facing the multiple peer influences for engaging in transgressive conduct [20].

### Self-efficacy: Measurement and multidimensionality

According to self-efficacy theory, in generating scales measuring efficacy beliefs, scales must be tailored to the different aspects of children’s life [2, 21]. In conceptualizing the development of instruments for the evaluation of Self-efficacy beliefs, Bandura [21] underlined the importance of identifying specific behavioural features that may be controlled by individuals and that sustain performances within a specific domain.

The Multidimensional Scales of Perceived Self-Efficacy (MSPSE; [22]) were developed by Bandura with the aim of measuring different domains relevant to children’s and adolescents’ life tapping nine different facets. Seven facets have been defined by Pastorelli and colleagues (2001) as follows: “a) *Self-efficacy for academic achievement* measures children beliefs in their capabilities to master different subject matters; b) *Self-efficacy for self-regulated learning* assesses children’s efficacy to structure environments conducive to learning and to plan and organize academic activities; c) *Self-efficacy for leisure and extracurricular activities* assesses children’ beliefs that they can carry out recreational and student group activities; d) *Self-regulatory efficacy* assesses children beliefs to resist peer pressure to engage in high-risk activities involving alcohol, drugs, and transgressive conduct; e) *Perceived social self-efficacy* assesses children beliefs in their capability to initiate and maintain social relationships and to manage interpersonal conflicts; f) *Self-assertive efficacy* measures children’s perceived capability to voice their opinions, to stand up to mistreatment, and to refuse unreasonable request; g) *Perceived self-efficacy to meet others’ expectations* measures children’s beliefs in their capability to fulfill what their parents, teachers, and peers expect of them, and to live up to what they expect of themselves.” (p.89 [23]). The two other facets are h) *Enlisting Parental and Community Support* and i) *Enlisting Social Resources* which measure children’s beliefs in their capacity to seek parents’, teachers’ and peers’ support in school activities [22].

Based on this original version, some studies have examined the factorial structure of MPSPE through exploratory factor analyses [24, 25], while a more limited number of studies tested the scale’s multidimensionality through a confirmatory approach (e.g. [26]). In Miller’s study [25] with North American high school students the nine dimensions were represented by three second-order factors that they labelled *Social Self-Efficacy*, *Task Management Efficacy*, and *Academic Efficacy*. In another study with North American college students [24] the multidimensionality of MSPSE was confirmed and represented by a three-factor structure. Finally, in a cross-national study, Pastorelli et al. [23] tested and replicated the MSPSE’s factorial structure in three countries- Italy, Poland and Hungary—in a sample of 1180 adolescents using Principal and Simultaneous component analyses. In this study, the two subscales related to enlisting parents’, teachers’ and community support were not included. Results revealed the existence of three basic factors underlying the seven facets: *Academic* self-efficacy, *Social* self-efficacy, and self-*Regulatory* efficacy. Authors found that *Academic* self-efficacy included those items designed to measure self-regulated learning efficacy, academic achievement self-efficacy, and self-efficacy to meet others’ expectations. *Social* self-efficacy included items related to self-assertiveness efficacy, and leisure-time skill and extracurricular activities efficacy. Finally, *Self-Regulatory* efficacy was defined by those items measuring self-regulatory efficacy to resist peer pressure. In addition, through Confirmatory Factor Analyses, Pastorelli & Basili [27] confirmed the three-factor structure on the Italian longitudinal sample of adolescents, that was found invariant across gender (see [14] for description of the sample).

More recently, Oliveira et al. [25] used the three subscales of MSPSE, that is self-efficacy for academic achievement, self-regulated learning, and leisure and extracurricular activities in a sample of Portuguese adolescents. CFAs showed the existence of a hierarchical model with 28 observed variables, three first-order and one second-order latent variables that were invariant across gender and children’s grades (5th and 6th grades).

Based on these last findings, we wanted to take a step forward in the validation of the MSPSE scale, not only by using the confirmatory approach on the total scale, but also by extending its examination to other cultural contexts. To our knowledge, no studies have examined the multidimensionality of MSPSE in Colombia and thanks to the collaborative effort of Italian and Colombian researchers over the last years [Italian and Colombian Investigators were involved in the dissemination in Colombia of a school-based program designed to promote children’s prosocial competences in school settings (CEPIDEA, Italian acronym for Promoting Emotional and Prosocial Skills to counteract Externalizing Problem in Adolescence, see [28]), we aimed to potentially contributing to the validity of these scales in the Colombian context and to make comparisons between Italy and Colombia. Measurement Invariance (MI) is a prerequisite for making meaningful comparisons between independent groups [29]. Specifically, through MI we examined the extent to which MSPSE demonstrated construct comparability across the two groups of Italian and Colombian adolescents and, therefore, rendered similarities and differences across groups interpretable.

In examining the presence of potential similarities and differences in adolescents’ Self-Efficacy appraisals in the two cultural contexts—Italy and Colombia—we might refer to Hofstede's definition of culture [30] as "the collective programming of the mind which distinguishes the members of one human group from another" (p. 25) and to his two macro indicators, namely *Individualism/Collectivism* and *Power Distance*. Regarding Italy and Colombia, Italian culture is characterized by higher levels of *Individualism* as compared to the Colombian one, while there are less differences in the *Power/Distance* indicator [31]. Overall, we think there is no reason to believe that individuals from collectivist cultures appraise less their efficacy beliefs, goals and personal accomplishments. Likely, the *Power/Distance* dimension could affect more the efficacy appraisal, being related to normative relationships within specific contexts [32].

We focused on the school context and according to Hofstede’s Indicators both countries are characterized by high levels of *Power/Distance*—with Colombia slightly higher than Italy- meaning that, at school, the relationships between teachers and students are more likely to be characterized by similar hierarchical order, in which teacher and student role are hierarchical, fixed and not negotiable. Based on this general assumption we are inclined to think that we cannot formulate strong and clear hypotheses regarding differential functioning of the MSPSE scale scores between Italy and Colombia, although we could reasonably expect strong similarities across the two samples.

With regards to the role of gender, some research suggests that Self-Efficacy beliefs may differ in important ways by gender, though the findings are somewhat inconsistent [33]. Pastorelli et al. [23] found that adolescent females report higher levels of global academic self-efficacy than their male peers. In a metanalysis conducted on 247 samples by Huang [34] males were found to have slightly higher levels of academic self-efficacy overall as compared to their female peers. However, when considering specific subject areas, females had greater self-efficacy for language and arts whereas males had higher self-efficacy for computer and social sciences. Similar findings have been shown regarding *Self-regulatory Self-efficacy*: girls reported themselves as more capable to resist peer pressure in engaging in transgressive activities and behaviors (e.g. [20, 23, 35]). When considering *Social-Self efficacy*, findings are less convergent in showing gender differences. Whereas Bandura et al. [2] found that girls reported lower capability in social relations and activities, other studies did not evidence gender differences (e.g. [20, 35]). Exploring these gender differences is important due to the implications and the relationship between self-efficacy and academic and social outcomes ([8, 36]).

The present study is the first to test the factorial structure and the MI of MSPSE scale both across gender and nationality. Our purpose was twofold. First, we aimed to test, through CFAs, the multidimensional structure of the MSPSE scale in Italy and Colombia, separately. Second, we tested the extent to which the original three factors solution found by previous studies, were invariant both across Italian and Colombian adolescents and across gender. The study was intended to validate the assessment of Self-efficacy beliefs as multifactorial construct and to extend cross-cultural validity of MSPSE by testing the entire scale in culturally diverse samples.

## Method

### Participants

IRBs at Sapienza University of Rome, Universidad del Magdalena, Universidad de Manizales and Universidad de San Buenaventura-Medellin approved the study protocol. Written teachers', parental informed consent and child assent were obtained.

#### Italian sample

The Italian participants were 564 students ranging in age from 12 to 13 years (M = 12.52 years; SD = 0.50; 52,1% male) from Genzano, a residential community near Rome. The youth were drawn from 7^th^- 8^th^ grades of two middle schools and they were from middle-class families. All participants lived with their parents and were of Italian origin.

#### Colombian sample

A total of 645 students attending the 7^th^ grade (M = 12.36 years; SD = 0.48; 52,9% male) participated at the study. The total sample was drawn from 3 Colombian sites: Manizales, Medellin and Santa Marta. Participants were involved in a school-based intervention project called CEPIDEA (see [28]), designed to promote prosocial behaviors in early adolescence within the classroom setting. For the purpose of the present study, we considered the baseline dataset, prior to the intervention.

### Procedure

For both samples, letters describing the study were sent home via children and parental informed consent was obtained for all students at each site. Questionnaires for students were administered in each classroom by trained members of the research team during school hours (see [28] for the detailed description).

### Measures

In the present study we used the adolescents’ version of the Multidimensional Scales of Perceived Self-Efficacy (MSPSE, [9, 23]) composed of 37 items representing three domains of functioning. From the original scale, we were able to compare 33 out of the 37 items, due to the different purposes and instruments’ selection of the studies from which the Italian and Colombian samples were drawn. The scale was translated in Spanish and then back-translated by bilingual experts. The three domains addressed by the scale represent adolescents’ functioning during classes in their schools:

- *Perceived Academic Efficacy* included 16 items tapping different domains of academic activities: 4 items assessed children’s beliefs in their capability to master different curricular areas (e.g. mathematics, sciences); 10 items assessed perceived efficacy for regulating one’s own motivation and learning activities and 2 items measured efficacy beliefs to parental and teacher expectations. Cronbach’s alphas were .87 (*Italy*) and .89 (*Colombia*).
- *Perceived Social Efficacy* included 12 items tapping different social domains: leisure and extracurricular group activities; children’s beliefs to form and maintain social relationships and to manage interpersonal conflicts; self-assertive efficacy. Cronbach’s alphas for the two samples was .79 (*Italy*) and .86 (*Colombia*).
- *Self-Regulatory Efficacy* included 5 items that measured children’s capability to resist peer pressure to engage in high-risk activities such as alcohol and transgressive activities that can get them into trouble. Cronbach’s alphas for the Italian sample was .65 and .78 for the Colombian sample.

For each item, participants rated their belief in their level of capability to execute the designed activities using a 5-point scale ranging from 1 (*cannot do at all*) to 5 (*highly certain can do*).

### Data analysis

Preliminarily, items’ descriptive statistics were estimated and Exploratory Factor Analyses (EFAs) with oblimin rotation were conducted on the 33 items. Analyses were conducted in *Mplus* (v.7, [37]). The MSPSE items were considered ordered categorical variables because of the four-point response format and the recommended robust weighted least squares (WLSMV) estimator was used [37]. When indicators are categorical, the parameters of interest in invariance testing are factor loadings and thresholds, intended as cutoffs that define the ranges within each observed categorical response value are selected [29].

First, in order to test the three-factor structure of the MSPSE scale, Confirmatory Factor Analysis (CFAs) models were performed separately by country and gender. Then a Multigroup Confirmatory Factor Analysis (MGCFA) was implemented to test the hypotheses regarding MI across country and gender. A model-fitting process was implemented following the review by Vandenberg and Lance [29] and three nested models were tested in the following order: configural, metric and scalar. To test for *configural invariance*, the manifest variable intercepts/thresholds, residual variances, and factor loadings were freely estimated for all groups. To test for *metric invariance*, loadings were constrained to be equal across groups for all indicators. Finally, in order to test *scalar invariance*, variables’ intercepts/thresholds were constrained to be equal across groups. If invariance restriction did not hold for all items *(partial invariance)*, the inspection of modification indices (MIs) was used to identify the noninvariant items and to refine the structural models [38]. Each form of invariance was nested in the previous model and χ^2^ difference test was performed through *Mplus* DIFFTEST function to compare nested models. Comparative Fit Index (CFI) and the root mean square error of approximation (RMSEA) were used to evaluate models’ goodness-of-fit. CFI values greater than .90 [39] and RMSEA values smaller than .07 [40] were considered acceptable. As a further test of goodness-of-fit, following the procedures described by MacCallum et al. [41], for each CFA model we also examined the 90% confidence interval power estimates for RMSEA in relation to tests of a "close fit" (RMSEA = .05 vs. .08). Power is defined as the probability of not rejecting the null hypothesis, therefore a close fit of the study sample covariance matrix with the covariance matrix implied by the models in our study [42].

Furthermore, because the *χ2* is sensitive to sample sizes, ΔCFI and ΔRMSEA, which are more powerful in detecting lack of invariance in large sample sizes [43], were used to interpret which step of measurement was most tenable for the data. MI was considered established when model comparisons showed ΔCFI ≤ .010 and ΔRMSEA ≤ .015 [43, 44].

## Results

### Descriptive statistics and preliminary analysis

Means, standard deviations (SD), range (minimum and maximum), skewness and kurtosis were calculated for each of the 33 items of MSPSE in the total sample (Table 1). Values less than 2 for univariate skewness and less than 5 for univariate kurtosis were used as criteria for evaluating univariate normality [45]. With the exception of few items (16, 21, 22, 33) in the Italian sample, results showed satisfactory values for both skewness and kurtosis. Preliminary Exploratory Factor Analyses conducted separately on Colombian and Italian samples showed that most of the items had main loadings on the intended factors, with the exception of few items. Regarding *Perceived Academic Efficacy*, item 15, which refers to self-efficacy beliefs to participate in group discussions in class, showed higher loadings on *Perceived Social Efficacy* both in the Italian and Colombian samples. Regarding *Perceived Social Efficacy*, items 25 and 29 in the Italian sample–fulfil self-expectations and work in groups—and items 23 in the Colombian sample—fulfil parents’ expectations—had higher loading on *Perceived Academic Efficacy*. No cross-loadings were found in the dimension of *Self-regulatory efficacy*.

**Table 1:** Descriptive statistics for the three self-efficacy components shown separately by national origins.

Sites	Italy (N = 564)				Colombia (N = 645)			
Item	Mean	Sd	Skewness	Kurtosis	Mean	Sd	Skewness	Kurtosis
***Academic Efficacy***		α = .87			α = .89			
*Item 1*	3,90	1,13	-1,06	0,11	3,01	1,42	0,08	-1,51
*Item 2*	4,09	0,94	-1,36	1,58	3,53	1,25	-0,58	-1,00
*Item 3*	3,93	1,03	-1,06	0,30	3,36	1,31	-0,28	-1,37
*Item 4*	4,05	1,00	-1,26	1,00	3,67	1,28	-0,77	-0,71
*Item 5*	4,19	0,96	-1,43	1,56	3,47	1,40	-0,59	-1,10
*Item 6*	3,66	1,16	-0,67	-0,81	3,19	1,30	-0,07	-1,47
*Item 7*	3,62	1,14	-0,66	-0,82	3,26	1,35	-0,17	-1,45
*Item 8*	3,32	1,33	-0,39	-1,27	3,35	1,36	-0,32	-1,37
*Item 9*	4,34	0,87	-1,79	3,47	3,56	1,38	-0,57	-1,13
*Item 10*	4,13	0,86	-1,35	1,90	3,67	1,25	-0,74	-0,74
*Item 11*	4,09	0,89	-1,31	1,57	3,48	1,31	-0,45	-1,22
*Item 12*	4,07	0,94	-1,31	1,38	3,52	1,30	-0,47	-1,21
*Item 13*	3,98	1,09	-1,08	0,17	3,11	1,44	-0,07	-1,52
*Item 14*	3,90	1,13	-1,33	1,57	3,56	1,28	-0,59	-1,00
*Item 23*	4,03	0,97	-1,26	1,09	3,85	1,31	-1,01	-0,31
*Item 24*	4,00	0,92	-1,28	1,36	3,70	1,29	-0,76	-0,76
***Social Efficacy***		α = .79			α = .86			
*Item 15*	3,99	1,07	-1,09	0,21	3,18	1,40	-0,13	-1,48
*Item 16*	4,51	0,78	-2,24	6,03	3,96	1,27	-1,10	-0,08
*Item 17*	4,34	0,87	-1,73	3,15	3,92	1,29	-0,97	-0,44
*Item 18*	4,29	0,95	-1,67	2,44	3,67	1,45	-0,70	-1,05
*Item 25*	4,27	0,93	-1,54	1,94	3,83	1,24	-0,98	-0,26
*Item 26*	4,02	1,21	-1,17	0,16	3,80	1,34	-0,91	-0,56
*Item 27*	4,10	1,11	-1,29	0,69	3,88	1,27	-0,98	-0,35
*Item 28*	4,25	0,93	-1,57	2,28	3,53	1,32	-0,49	-1,19
*Item 29*	4,39	0,81	-1,82	3,94	3,92	1,21	-1,11	0,11
*Item 30*	4,04	1,00	-1,22	0,83	3,57	1,31	-0,58	-1,05
*Item 31*	4,42	0,83	-1,80	3,37	3,88	1,22	-1,02	-0,15
*Item 32*	4,30	0,89	-1,76	3,31	3,48	1,32	-0,52	-1,13
***Self-Regulatory Efficacy***		α = .65			α = .78			
*Item 19*	3,89	1,30	-0,95	-0,47	2,97	1,43	0,06	-1,51
*Item 20*	3,72	1,36	-0,73	-0,92	3,14	1,49	-0,13	-1,54
*Item 21*	4,51	1,13	-2,30	3,83	3,29	1,68	-0,31	-1,64
*Item 22*	4,51	1,09	-2,25	3,70	3,30	1,67	-0,30	-1,65
*Item 33*	4,48	0,95	-2,25	4,63	3,38	1,50	-0,36	-1,45

### Confirmatory factor analyses

First, in order to test the MSPSE three-factor structure, CFAs were conducted on the total sample and separately by country (*Italy* and *Colombia*). The three-factor model was found to provide acceptable fit for the total sample [χ ^2^ (492) 2764.126, *p* < 0.01, *RMSEA =* .06 (90% CIs = .060–.064), *CFI =* .93] and separately for Italy and Colombia (Table 2). Furthermore, based on tests proposed by MacCallum et al. [41], confidence intervals about RMSEA for each CFA model were consistent with the extremely high-power estimates, all greater than .999 (Table 2), demonstrating the high precision of the estimate of fit.

**Table 2:** Tests for invariance of self-efficacy for MSPSE measurement model across gender and country: Summary of goodness-of-fit statistics.

Measurement Invariance across Gender												
Italy												
				RMSEA CI and Power								
	χ ^2^	*df*	CFI	RMSEA	Lower	Upper	Power	Model Comparison	χ^2^_diff_	Δ*df*	Δ*CFI*	Δ*RMSEA*
Male (n = 294)	945.125	492	.910	.056	.051	.061	>.999					
Female (n = 271)	828.443	492	.920	.050	.044	.056	>.999					
Model 1. Configural Invariance	1767.152	984	.916	.053	.049	.057						
Model 2. Metric Invariance	1663.416	1014	.931	.048	.043	.052		2 vs 1	32.760	30	.015	-.005
Model 3. Scalar Invariance	1811.247	1077	.921	.049	.045	.053		3 vs 2	235.292*	63	-.010	-.001
Model 4. Partial Scalar Invariance^a^	1797.293	1076	.923	.049	.045	.053		4 vs 2	209.150*	62	-.008	.001
Colombia												
					RMSEA CI and Power							
	χ ^2^	*df*	CFI	RMSEA	Lower	Upper	Power	Model Comparison	χ^2^_diff_	Δ*df*	Δ*CFI*	Δ*RMSEA*
Male (n = 341)	882.649	492	.955	.048	.043	.053	>.999					
Female (n = 304)	1073.371	492	.920	.062	.057	.067	>.999					
Model 1. Configural Invariance	1969.173	984	.938	.056	.052	.059						
Model 2. Metric Invariance	1775.735	1014	.952	.048	.045	.052		2 vs 1	30.547	30	.014	-.008
Model 3. Scalar Invariance	1871.973	1077	.950	.048	.044	.051		3 vs 2	131.008*	63	-.002	.000
Measurement Invariance Across Country												
					RMSEA CI and Power							
	χ ^2^	*df*	CFI	RMSEA	Lower	Upper	Power	Model Comparison	χ^2^_diff_	Δ*df*	Δ*CFI*	Δ*RMSEA*
Italy (n = 564)	1502.969*	492	.904	.060	.57	.064	>.999					
Colombia (n = 645)	1743.048*	492	.926	.063	.060	.066	>.999					
Model 1. Configural Invariance	3223.187*	984	.920	.061	.059	.064						
Model 2. Metric Invariance	3166.358*	1014	.923	.059	.057	.062		2 vs 1	170.615*	30	.003	-.002
Model 3. Scalar Invariance	3759.10*	1728	.906	.063	.061	.065		3 vs 2	894.920*	96	-.017	.004
Model 4. Partial Scalar Invariance^b^	3528.509*	1103	.914	.060	.058	.063		4 vs 2	554.634*	89	-.009	.001

*Notes*: χ ^2^ = Chi-square Goodness of Fit; *df* = degrees of freedom; CFI = Comparative Fit Index; RMSEA = Root Mean Square Error of Approximation. Upper and lower limits for the observed RMSEA confidence intervals (RMSEA CI) and power estimates for a "close fit" (RMSEA = .05 vs. .08) are those specified by MacCallum et al. (1996). All the Δ index comparisons are made with respect to the previous model.

* p < = .01

^a^ Free threshold of Perceived Social Efficacy: item 26, free threshold 1.

^b^ Free thresholds of Perceived Academic Efficacy: item 1, threshold 2; item 7, threshold 3; item 8, threshold 3; item 9, threshold 2; item 23, threshold 3; item 24, threshold 3.

Free threshold of Perceived Self-Regulatory Efficacy: item 32, threshold 2.

Then, we proceeded through the steps of testing measure invariance across country and gender.

#### MGCFA across country

All fit indices and statistical tests are presented in Table 2. Regarding country invariance, the model with factor loadings unconstrained to be equal across countries fit the data reasonably well, confirming configural invariance [χ^2^ (984) = 3223.187, p < .01, CFI = .920, RMSEA = .061(.059,.064)]. After that, the hypothesis of full metric invariance was tested and accepted [Δχ^2^ (30) = 170.615, p < .001, ΔCFI = .003, ΔRMSEA = -.002], supporting that the scale structure and the factor loadings were equivalent for the Italian and Colombian samples. Next, full scalar invariance was tested and rejected, because the model did not show a good fit and differed significantly from the metric model. Then, as suggested by the modification indices, we allowed 7 thresholds to vary freely (see Table 2) in order to test for partial scalar invariance: the 7 released thresholds referred to 6 items from the *Perceived Academic Efficacy* factor (i.e. “*learn general mathematics*?”, “*concentrate on school subjects*?”, “*take class notes of class instruction*?”, “*use the library to get information for class assignments*?”, “*live up to what your teachers expect of you*?*”*, “*live up to what you expect of yourself*?”) and one item from the *Perceived Social Efficacy* (i.e. “*deal with situations where others are annoying you or hurting your feeling*s?”).

**Fig 1:**
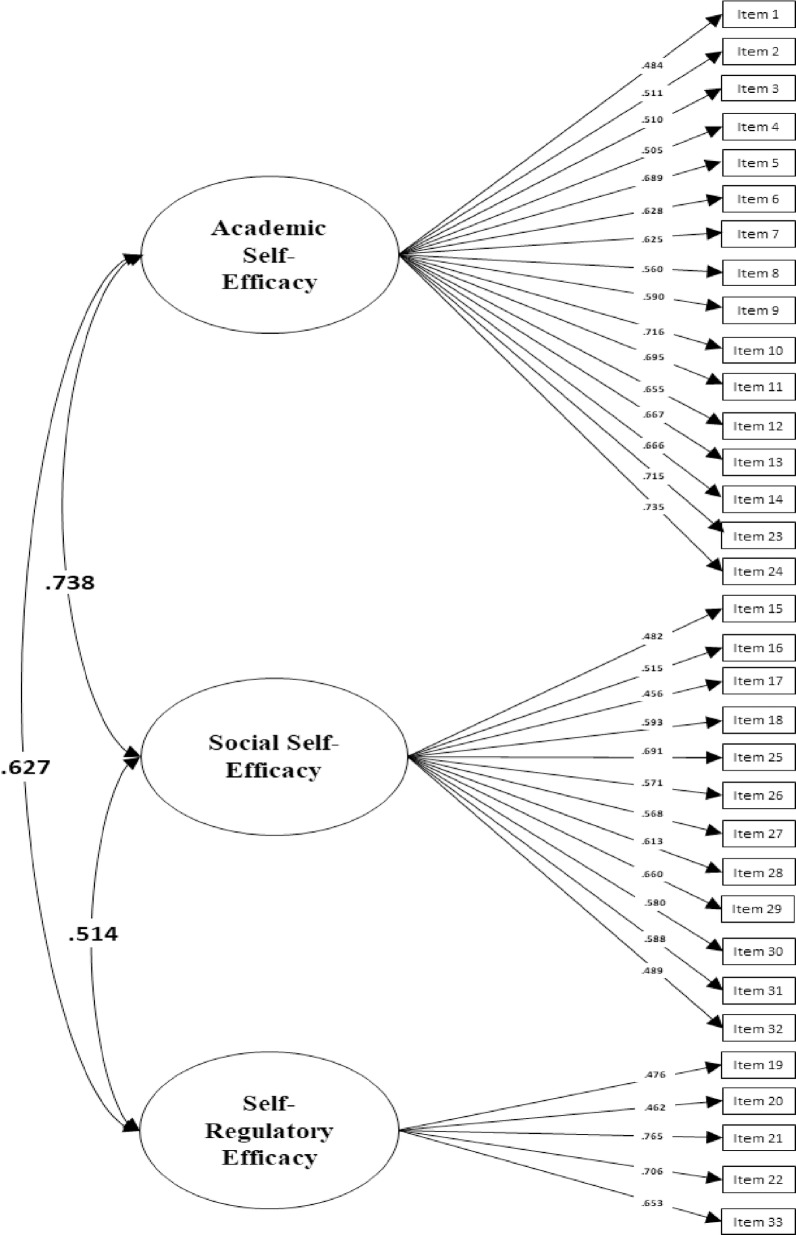
Factor loadings of the MSPSE for Italian adolescents.

**Fig 2:**
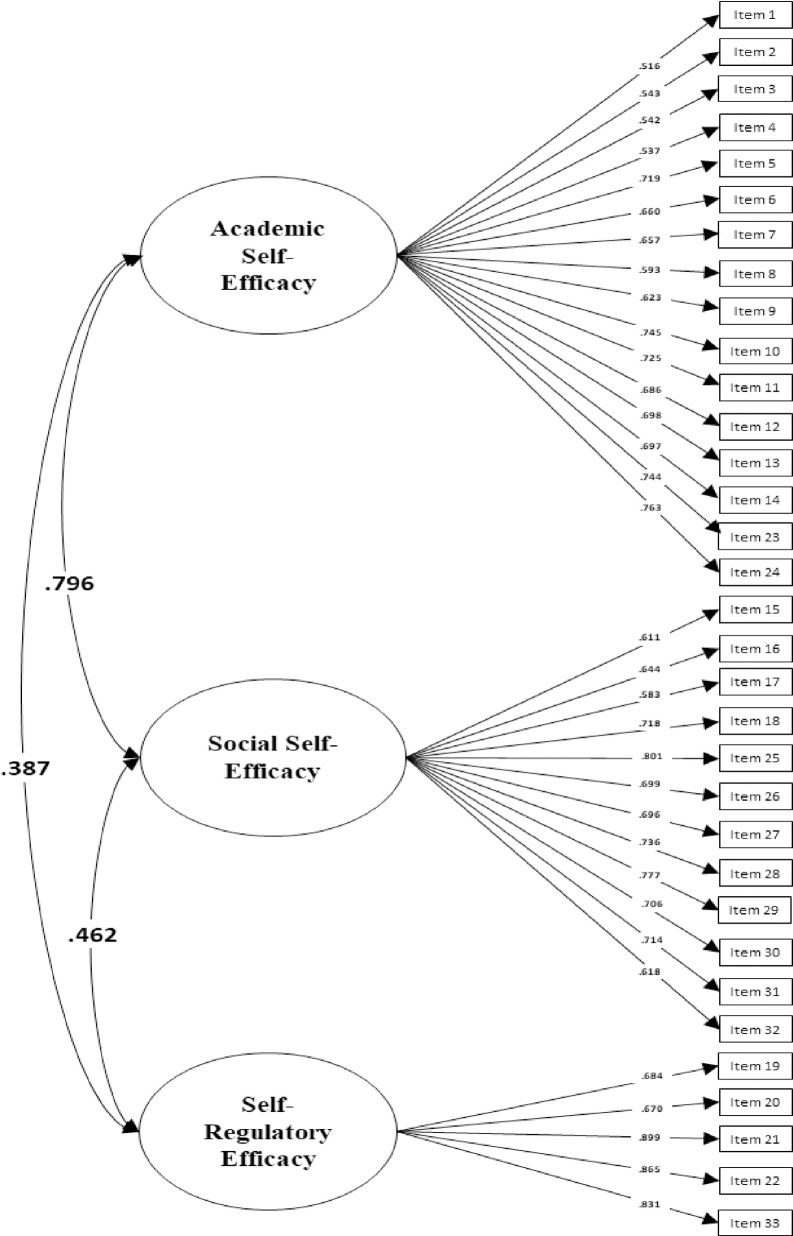
Factor loadings of the MSPSE for Colombian adolescents.

After these thresholds were freed, the model provided acceptable fit change [ΔCFI = -.009, ΔRMSEA = -.001] and showed that partial scalar invariance could be established. Although full invariance was not reached for the thresholds of some items, reaching partial scalar invariance equally supported that *Academic*, *Social* and *Regulatory* dimensions are measured in similar ways across the two countries (Fig 1 and Fig 2.).

Once partial scalar invariance was established, it was possible to explore possible differences at the level of the latent means of the three factors. Results showed that, on average, Colombian students perceived themselves significantly less efficacious in the three factors compared to the Italian students (*z* score *=* -10.6, p < .001; *z* score *=* -9.8, p < .001; *z* score *=* -13.2, p < .001).

#### MGCFA across gender

In order to test for invariance across boys and girls, MGCFAs were conducted separately for the Italian and Colombian samples. Regarding gender invariance, the model with factor loadings not constrained to be equal across gender fit the data reasonably well, confirming configural invariance in Italy and Colombia. After that, also the hypothesis of full metric invariance was tested and supported in both countries. (Table 2). Next the hypothesis of full scalar invariance was tested, but full scalar invariance was established for Colombian boys and girls [Δχ2 (30) = 30.547, ns, ΔCFI = .014, ΔRMSEA = -.008; Δχ2 (63) = 131.008, p < .001, ΔCFI = -.002, ΔRMSEA = -.000], while in the Italian sample the full scalar invariance model did not fit well with the data and was significantly worse than the previous metric model (Table 2). Modification indices suggested to free item 26’s threshold (*“make and keep female friends”*) belonging to the perceived *Social Efficacy* dimension, and the resulting model provided an acceptable fit change [ΔCFI = -.008, ΔRMSEA = -.001], showing that partial scalar invariance could be established between Italian boys and girls.

Latent mean comparisons showed that, on average, Italian girls rated themselves significantly higher in *Perceived Academic Efficacy* (*z* score *=* 3.1, p < .001) and *Self-Regulatory Efficacy* (*z* score *=* 2.6, p < .05) than boys. No significant latent mean differences were found between Colombian boys and girls.

## Discussion

The MSPSE scale is a widely used self-report measure to assess youth’s Self-Efficacy beliefs in academic and social contexts. To date, there have been very few studies reporting the factorial structure and multidimensionality of the Multidimensional Scales of Perceived Self-Efficacy (MSPSE) developed by Bandura [22] and to our knowledge no study has tested the MSPSE scale’s cross-national Measurement Invariance. The aim of the present study was to test the measurement invariance of MSPSE scale comparing two samples of adolescents from Italy and Colombia. In particular, we were interested in testing invariance both across country and gender. Examining whether the *Academic*, *Social* and *Regulatory* Self-Efficacy dimensions are invariant cross-culturally will allow researchers and professionals to use and interpret their results appropriately.

CFAs conducted on the total sample and separately for Colombia and Italy confirmed the MSPSE scale’s three-factor structure as identified by previous validation studies (e.g. [23]). Multigroup CFAs conducted between the two countries supported measurement invariance: full configural, metric and partial scalar invariance validated MSPSE scale comparability among Italian and Colombian adolescents. Partial scalar invariance was reached after 7 thresholds were freed, showing that for some CPSE’s items, the score’s probability to be assigned to a particular categorical rating differed across country. To note, partial scalar invariance was mostly referred to those items regarding *Perceived Academic Efficacy*. Specifically, Italian students respond more frequently on higher response categories (i.e. “quite well” and “very well”) than Colombian students on item 7 “Always concentrate on school subjects during class”, item 8 “Take good notes during class instruction”, item 23 “Live up to what my parents expect of me” and item 24 “Live up to what my teachers expect of me”. On the other hand, Colombian students had higher probability to respond on the lower response categories (i.e. “not too well”) on item 1 “Learn general mathematics” and item 9 “Use the library to get information for class assignments”. Explanations for partial-invariance in the domain of perceived Academic Efficacy may be found in the different perceptions and beliefs that students may hold regarding the school contexts and practices in their countries [46, 47]. Recent reports about Latin American countries raised some concerns on the development and achievements of their educational systems [48, 49]. Academic success is influenced by a variety of factors or conditions such as financial or material resources, curriculum, abilities of the students involved, teachers and the external agents interested in the quality of education (such as parents). According to Social Cognitive Theory, the environment and the social systems influence human behavior through psychological mechanisms of the Self-system. These contextual factors may influence student's aspirations, self-efficacy beliefs, personal standards and emotional states hampering their learning opportunities and challenging their perception of efficacy to exert control over their academic and social development. Findings of partial invariance in the response categories of some items, may reflect different expectations and perceptions of Colombian students regarding their school achievement and suggested that the MPSE scale is well suited to reliably test different components of academic efficacy also in a cross-cultural framework.

However, even if the full measurement invariance was not obtained, results allow appropriate cross-group comparisons [29]. Therefore, these findings imply that in Italy and Colombia, the MSPSE scale presents the same structure, with the majority of items being equally associated to *Academic*, *Social* and *Regulatory* Self-efficacy’s dimensions.

The establishment of scalar invariance across countries indicated that the mean differences of the MSPSE can be directly compared. Latent mean comparisons between the two countries showed that Italian students perceived themselves significantly more efficacious in the three dimensions. Once asked about their capability to regulate their own learning, to be socially involved at school and to resist peer pressure, Italian adolescents reported to feel more efficacious compared to their Colombian counterparts. Differences between countries were also found by Pastorelli et al. [23] when Italian children judged themselves more academically efficacious than Hungarian children and more socially efficacious than both Hungarian and Polish children. To our knowledge, there are no previous studies comparing Italian and Colombian adolescents. As mentioned above for item’s partial invariance, differences in perceived efficacy may reflect differences in national educational systems and practices between Italy and Colombia. As recently highlighted by OECD reports [48], one of the major concerns for the Latin America’s and the Caribbean educational systems is that young people are provided with basic education models whose level can hardly be compared with the performance levels of the youth from more developed countries, as can be seen by reviewing the results of the PISA tests [49, 50, 48]. Colombian review of educational policies [48] showed that, though improving, access to educational facilities from early-adolescence is still difficult for Colombian students that “struggle to make adequate progress, repeat grades or drop out altogether. With 41% of 15-year-olds having repeated at least a year, Colombia makes much greater use than most countries of this ineffective and costly practice” (p. 8 [48]). These contextual factors may have produced some disparities compared to Italy where, even though several regional differences are still present, educational indicators such as school drop-out have been slightly improving due to the development of new programs [51]. Further studies are needed in order to better explain the potential source of cross-national differences and their effect on Self-efficacy beliefs which may allow us to make appropriate comparisons among students across countries.

Regarding gender invariance, results showed, especially for Colombian students, the presence of full scalar invariance, evidencing that items of perceived Self-Efficacy load on the same factors that are defined in the same way across gender and that boys and girls reported similar levels of *academic*, *social* and *regulatory* perceived Self-efficacy.

For the Italian sample, we found support for partial scalar invariance by freeing the first threshold of item 26 (*“make and keep female friends”*). Evidence of partial scalar invariance shows the presence of differences at the level of item’s thresholds indicating that Italian girls, when asked about their capability to make and keep female friends, were more likely to select a higher categorical response than boys.

Finally, latent means comparisons evidenced no differences in any of the three dimensions between Colombian boys and girls. Differently, in the Italian students, girls perceived themselves significantly more efficacious in academic activities and in resisting to peer pressure than did boys while no gender differences were found in social perceived efficacy. Accordingly, other studies have found that girls showed a higher sense of academic efficacy and were favoured in self-efficacy for self-regulated learning compared to boys [52, 53]. A study from Zimmerman and Martinez-Pons [54] with students in Grades 5, 8, and 11, evidenced that girls displayed more goal-setting and planning strategies, and they kept records and self- monitored more frequently than did boys.

Regarding the Colombian sample, we did not find gender differences in any of the three dimensions, showing that Colombian boys and girls reported similar beliefs on their *Academic*, *Social* and *Regulatory* efficacy. To our knowledge, the present study is the first to test gender invariance on MSPSE scale in a Colombian sample and future studies are needed to investigate potential gender differences in the three dimensions.

Given the importance of perceived Self-efficacy beliefs for adolescent development, we underline the relevance of MSPSE scale and its validation both across cultures and gender. Test of factorial validity and measurement invariance of the MSPSE scale have several implications for the field of education. We believe that results of the current research will have practical significance for school administrators, educators and researchers in developing their understanding of self-efficacy and the influence that it has on academic achievement. MSPSE scale represents a useful measure to be included in intervention and educational programs that need to detect potential behavioural risk factors and prevent school-failure [28]. The new challenges that students constantly face at school required them to develop beliefs in their capabilities and the ability to self-regulate their own learning. Moreover, perceived self-efficacy operates on academic functioning also by raising academic aspirations and self-evaluative standards for the quality of their academic work [23]. Educators need to develop an understanding of methods and practices for promoting their students’ self-efficacy who have previously showed low academic achievement and poor social relations with their peers [9, 12]. In fact, as evidenced by other studies, these self-efficacy dimensions protect adolescents from risky behaviors, such as substance use, delinquency and favour academic and social adjustment [55, 56]. It is highly relevant to examine educational implications also on a cross-national perspective. Cross-cultural studies provide fundamental information about potential differences or similarities in students’ academic self-development. Given that much learning occurs outside of schools, professionals and researchers should be aware of culture-specific factors that influences self-efficacy beliefs in terms of accessibility and resources.

Finally, it is important to note some limitations of our results. The samples cannot be considered totally representative of the populations of the two countries although age, gender and level of education were matched and comparable. The results of our study should be then taken with caution. Further studies are needed to evaluate the generalizability of findings about partial scalar invariance obtained both regarding gender in the Italian sample and across country. In addition, future studies should consider relevant outcomes related to the three Self-Efficacy dimensions, such as academic achievements, risky and adaptive social behaviors, in order to further support their construct validity.

Nevertheless, taken together, these analyses indicate that Colombian and Italian students’ reports on MSPSE scale can be meaningfully compared. The three-factor structure of MSPSE is consistent with previous validation studies and confirm self-conception of efficacy as a multifaceted construct consistent across gender and countries. Acknowledging the psychometric properties of the MSPSE along with its strong theoretical and empirical bases, these results have the potential to add evidence to the structural validity of this scale with respect to its Italian and Colombian adaptation.

## Supporting information

S1 Data Data included in the paper. (XLS)

S1 Scales MSPSE Italian, Spanish and English version. (DOCX)

## References

[1] Bandura A. Social foundations of thought and action: A social cognitive theory. Englewood Cliffs, NJ: Prentice-Hall; 1986.

[2] Bandura A. Self-efficacy: The exercise of control. New York, NY US: Freeman; 1997.

[3] Grusec JE, & Hastings PD, editors. Handbook of socialization. New York, NY: Guilford Press; 2015.

[4] Schunk DH, & Zimmerman BJ. Influencing children’s self-efficacy and self-regulation of reading and writing through modeling. Reading and Writing Quarterly. 2007; 23: 7–25.

[5] Pajares F, & Urdan T, editors. Self-efficacy beliefs of adolescents. Greenwich, CT: Information Age Publishing; 2006.

[6] Gaskill PJ, & Hoy AW. Self-efficacy and self-regulated learning: The dynamic duo in school performance In: Aronson J., editor. Improving academic achievement: Impact of psychological factors on education. San Diego, CA, US: Academic Press; 2002 pp. 185–208.

[7] Pajares F. Self-efficacy beliefs in academic settings. Review of Educational Research. 1996; 66: 543–578.

[8] Bandura A. Adolescent development from an agentic perspective In: Pajares F & Urdan T, editors. Self-efficacy beliefs of adolescents. Greenwich, CT: Information Age Publishing 2006; 5: 1–43.

[9] Bandura A, Barbaranelli C, Caprara GV, & Pastorelli C. Multifaceted impact of self‐efficacy beliefs on academic functioning. Child Development. 1996; 67(3: 1206–1222. 8706518

[10] Fernandez-Rio J, Cecchini JA, Méndez-Gimenez A, Mendez-Alonso D, & Prieto JA. Self-regulation, cooperative learning, and academic self-efficacy: Interactions to prevent school failure. Frontiers in Psychology. 2017; 8, 22 10.3389/fpsyg.2017.00022 28154544 PMC5243853

[11] Bandura A, Caprara GV, Barbaranelli C, Pastorelli C, & Regalia C. Sociocognitive self-regulatory mechanisms governing transgressive behavior. Journal of Personality and Social Psychology. 2001; 80(1): 125–135. 11195885

[12] Carroll A, Houghton S, Wood R, Unsworth K, Hattie J, Gordon L, & Bower J. Self-efficacy and academic achievement in Australian high school students: The mediating effects of academic aspirations and delinquency. Journal of Adolescence. 2009; 32(4): 797–817. 10.1016/j.adolescence.2008.10.009 19027942

[13] Schunk DH. Self-regulation of self-efficacy and attributions in academic settings In: Schunk DH & Zimmerman BJ, editors. Self-regulation of learning and performance: Issues and educational applications. Hillsdale, NJ: Erlbaum; 1994 pp. 75–99.

[14] Caprara GV, Fida R, Vecchione M, Del Bove G, Vecchio GM, Barbaranelli C et al Longitudinal analysis of the role of perceived self-efficacy for self-regulated learning in academic continuance and achievement. Journal of Educational Psychology. 2008; 100(3): 525–534.

[15] Caprara GV, Alessandri G, & Eisenberg N. Prosociality: The contribution of traits, values and self-efficacy beliefs. Journal of Personality and Social Psychology. 2012; 102: 1289–1303. 10.1037/a0025626 21942280

[16] Caprara GV, Gerbino M, Paciello M, Di Giunta L, & Pastorelli C. Counteracting depression and delinquency in late adolescence: The role of regulatory emotional and interpersonal self-efficacy beliefs. European Psychologist. 2010; 15(1): 34–48.

[17] Hermann KS, & Betz NE. Path models of the relationships of instrumentality and expressiveness, social self-efficacy, and self-esteem to depressive symptoms in college students. Journal of Social and Clinical Psychology. 2006; 25(10): 1086–1106.

[18] Rocchino G, Dever B, Telesford A, & Fletcher K (2017). Internalizing and externalizing in adolescence: The roles of academic self -efficacy and gender. Psychology in the Schools. 2017; 54(9): 905–917.

[19] Allen JP, Leadbeater BJ, & Aber JL. The relationship of adolescents’ expectations and values to delinquency, hard drug use, and unprotected sexual intercourse. Development and Psychopathology. 1990; 2(1): 85–98.

[20] Caprara GV, Barbaranelli C, Pastorelli C, & Cervone D. The contribution of self- efficacy beliefs to psychosocial outcomes in adolescence: Predicting beyond global dispositional tendencies. Personality and Individual Differences. 2004; 37(4): 751–763.

[21] Bandura A. Guide for constructing self-efficacy scales In: Pajares F. & Urdan T, editors. Self-efficacy beliefs of adolescents. Greenwich, CT: Information Age Publishing; 2006 pp. 307–337.

[22] Bandura A. Multidimensional scales of perceived academic efficacy. Stanford, CA: Stanford University; 1990.

[23] Pastorelli C, Caprara GV, Barbaranelli C, Rola J, Rozsa S, & Bandura A. The structure of children's perceived self-efficacy: A cross-national study. European Journal of Psychological Assessment. 2001; 17(2): 87–97.

[24] Choi N, Fuqua DR, & Griffin BW. Exploratory analysis of the structure of scores from the Multidimensional Scales of Perceived Self-Efficacy. Educational and Psychological Measurement. 2001; 61: 475–489.

[25] Miller JW, Coombs WT, & Fuqua DR (1999). An examination of psychometric properties of Bandura’s multidimensional scales of perceived self-efficacy. Measurement and Evaluation in Counseling and Development. 1999; 31: 186–196.

[26] Oliveira ÍM, do Céu Taveira M, Porfeli EJ, & Grace RC. Confirmatory Study of the Multidimensional Scales of Perceived Self-Efficacy with Children. Universitas Psychologica. 2018; 17(4).

[27] Pastorelli C, & Basili E. La Scala per la Misura dell’Autoefficacia in Adolescenza: Struttura Fattoriale ed Invarianza di Misura (Project #C26A06YZY4). Rome, Italy: Centro Interuniversitario per la Ricerca sulla Genesi e lo Sviluppo delle Motivazioni Prosociali e Antisociali (CIRMPA); 2015.

[28] Caprara GV, Luengo Kanacri BP, Gerbino M, Zuffianò A, Alessandri G, Vecchio GM et al Positive effects of promoting prosocial behavior in early adolescence evidence from a school-based intervention. International Journal of Behavioral Development. 2014; 38: 386–396.

[29] Vandenberg RJ, & Lance CE. A review and synthesis of the measurement invariance literature: Suggestions, practices, and recommendations for organizational research. Organizational Research Methods. 2000; 3: 4–70.

[30] Hofstede G. Culture's consequences: International differences in work-related values. Beverly Hills, CA: Sage; 1980.

[31] Hofstede G, Hofstede GJ, & Minkov M. Cultures and organizations: Software of the mind Revised and Expanded (3^rd^ Edition). New York: McGraw-Hill; 2010.

[32] Hofstede G. Dimensionalizing Cultures: The Hofstede Model in Context. Online Readings in Psychology and Culture. 2009; 2.

[33] MacPhee D, Farro S, & Canetto SS. Academic self‐efficacy and performance of underrepresented STEM majors: Gender, ethnic, and social class patterns. Analyses of Social Issues and Public Policy (ASAP). 2013; 13(1): 347–369.

[34] Huang C. Gender differences in academic self-efficacy: A meta-analysis. European Journal of Psychology of Education. 2013; 28(1): 1–35.

[35] Vecchio GM, Gerbino M, Pastorelli C, Del Bove G, & Caprara GV. Multi-faceted self-efficacy beliefs as predictors of life satisfaction in late adolescence. Personality and Individual Differences. 2007; 43(7): 1807–1818.

[36] Deane KL, Harré N, Moore J, & Courtney MGR. The Impact of the Project K Youth Development Program on Self-Efficacy: A Randomized Controlled Trial. Journal of Youth and Adolescence. 2017; 46(3): 516–537. 10.1007/s10964-016-0463-9 26984753

[37] Muthén BO, & Muthén LK. Mplus user’s guide (7^th^ ed.). Los Angeles, CA: Muthén & Muthén; 2012.

[38] Steenkamp JEM, & Baumgartner H. Assessing measurement invariance in cross-national consumer research. Journal of Consumer Research. 1998; 25: 78–90.

[39] Kline RB. Principles and practice of structural equation modeling (3^rd^ ed.). New York, NY: Guilford; 2010.

[40] Browne MW, & Cudeck R. Alternative ways of assessing model fit In: Bollen KA, & Long LS, editors. Testing structural equation models. Newbury Park, CA: Sage; 1993 pp. 136–162.

[41] MacCallum R. C., Browne M. W., & Sugawara H. M. (1996). Power analysis and determination of sample size for covariance structure modeling. Psychological Methods, 1(2), 130.

[42] Schumacker R. E., & Lomax R. G. (2015). A Beginner’s Guide to Structural Equation Modeling (4th ed). New York, NY: Routledge.

[43] Cheung GW, & Rensvold RB. Evaluating goodness-of fit indexes for testing measurement invariance. Structural Equation Modeling. 2002; 9: 233–255.

[44] Chen FF. Sensitivity of goodness of fit indexes to lack of measurement invariance. Structural Equation Modeling. 2007; 14: 464–504.

[45] Curran PJ, West SG, & Finch JF. The robustness of test statistics to non-normality and specification error in confirmatory factor analysis. Psychological Methods. 1996; 1: 16–29.

[46] Restrepo F. C. (1996). Atlántida: una aproximación al adolescente escolar colombiano. Nómadas (Col), (4).

[47] González Ó. D. A. (2015). El contexto político en la construcción de ciudadanos en la escuela colombiana: un cuestionamiento al conflicto ya la distorsión de la educación. Ciencias Sociales y Educación, 4(8), 107–121.

[48] OECD. Education in Colombia, Reviews of National Policies for Education, OECD Publishing, Paris; 2016.

[49] Cajiao F. (2017). Educación superior en América Latina y el Caribe: desafíos y asuntos pendientes. *Revista Educación Superior y* Sociedad (ESS), 24(24), 161–180.

[50] Ramos R., Duque J. C., & Nieto S. (2016). Decomposing the rural-urban differential in student achievement in Colombia using PISA microdata. *Estudios de Economía Aplicada*, 34(2), 379–411

[51] dell’Istruzione, M. M. (2017). dell’Università e della Ricerca (2017). La dispersione scolastica nell’a.s. 2015/2016 e nel passaggio all’a.s. 2016/2017. MIUR–Ufficio Statistica e Studi

[52] Reskin BF (1991). Bridging the man back in: Sex differentiation and devaluation of women’s work In: Lorber J, & Farrel SA, editors. The social construction of gender. Newbury Park, CA: Sage; 1991. pp. 141–161

[53] Pajares F. Gender and perceived self-efficacy in self-regulated learning. Theory into Practice. 2002; 41(2): 116–125.

[54] Zimmerman BJ, & Martinez-Pons M. Student differences in self-regulated learning: Relating grade, sex, and giftedness to self-efficacy and strategy use. Journal of Educational Psychology. 1990; 82: 51–59.

[55] Bandura A. Social cognitive theory: An agentic perspective. Annual Review of Psychology. 2001; 52: 1–26. 10.1146/annurev.psych.52.1.1 11148297

[56] Caprara GV. (2002). Personality psychology: Filling the gap between basic processes and molar functioning In: von Hofsten C, & Bakman L, editors. Psychology at the turn of the millennium: Social, developmental and clinical perspectives. Hove, UK: Psychology Press; 2002 pp. 201–224.

