# Supplementary material for: Multidimensional Scales of Perceived Self-Efficacy (MSPSE): Measurement invariance across Italian and Colombian adolescents
Source: PLoS One. 2020 Jan 15;15(1):e0227756. doi: 10.1371/journal.pone.0227756 (PMC6961898; doi:10.1371/journal.pone.0227756)
Supplement: S1 Scales — (DOCX) [file pone.0227756.s002.docx]

**Supporting Information**

**MSPSE Spanish Version**

“Translation modified from [Pastorelli, C., Caprara, G. V., Barbaranelli, C., Rola, J., Rozsa, S., & Bandura, A. (2001)] under a CC BY license, with permission from [Prof. Concetta Pastorelli], original copyright [2001].

***En qué grado eres capaz de:***

#####

1. *Aprender la Matemáticas*
2. *Aprender el Lenguaje/Literatura*
3. *Aprender la Historia*
4. *Aprender las Ciencias Naturales*
5. *Finalizar las tareas o trabajos dentro del plazo fijado*
6. *Estudiar cuando aparecen otras cosas interesantes que hacer*
7. *Concentrarte en el estudio sin distraerte*
8. *Tomar apuntes de las explicaciones del profesor*
9. *Buscar información complementaria en la biblioteca, en Internet, etc.*
10. *Organizar tu tarea de clases*
11. *Planificar tu tarea de clases*
12. *Recordar la información presentada en clases o en los libros de texto*
13. *Fijar un lugar de estudio sin distracciones*
14. *Interesarte en las materias del colegio*
15. *Participar en los debates que se produzcan en clases*
16. *Aprender deportes*
17. *Hacer ejercicio físico*
18. *Aprender las habilidades necesarias para practicar deportes de equipo (por ej. baloncesto, fútbol…)*
19. *Resistir la presión de los amigos para hacer cosas que te pueden meter en líos*
20. *Resistir la tentación de faltar a clases cuando te aburres o te sientes desanimado*
21. *Resistir la presión de los amigos a fumar cigarrillos cuando tu no quieres*
22. *Resistir la presión de los amigos a beber alcohol en exceso*
23. *Conseguir lo que tus padres esperan de ti*
24. *Conseguir lo que tus profesores esperan de ti*
25. *Cumplir tus expectativas*
26. *Hacer y mantener amigas*
27. *Hacer y mantener amigos*
28. *Expresar tu opinión cuando en tu grupo de amigos se está discutiendo alguna cosa*
29. *Trabajar en grupo*
30. *Expresar tus opiniones cuando otros compañeros no están de acuerdo contigo*
31. *Defender tus derechos cuando piensas que te están tratando injustamente*
32. *Hacer frente a situaciones en que otros te están molestando o bromeando*
33. *Esquivar las peticiones insistentes de tus amigos a hacer algo prohibido*

**CPSE Italian Version**

“Translation modified from [Pastorelli, C., Caprara, G. V., Barbaranelli, C., Rola, J., Rozsa, S., & Bandura, A. (2001)] under a CC BY license, with permission from [Prof. Concetta Pastorelli], original copyright [2001].

Quanto sei capace di:

1. *imparare la matematica?*
2. *imparare l’Italiano?*
3. *imparare la Storia?*
4. *imparare le Scienze?*
5. *finire in tempo i compiti che ti sono stati assegnati a casa?*
6. *impegnarti nello studio quando hai altre cose interessanti da fare?*
7. *concentrarti nello studio senza farti distrarre?*
8. *prendere appunti delle spiegazioni dell’insegnante?*
9. *di fare ricerche che ti vengono assegnate utilizzando altri libri (che puoi trovare a casa, in biblioteca, etc.)*
10. *organizzarti nello svolgimento delle attività scolastiche?*
11. *programmare le tue attività scolastiche?*
12. *ricordare ciò che l’insegnante ha spiegato in classe e ciò che hai letto sui libri?*
13. *trovarti un posto dove studiare senza essere distratto?*
14. *interessarti alle materie scolastiche?*
15. *partecipare alle discussioni che avvengono in classe?*
16. *imparare nuovi sport?*
17. *Quanto sei bravo/a nelle normali attività di educazione fisica?*
18. *imparare ciò che serve per fare parte di una squadra sportiva (pallacanestro, pallavolo, ecc.)?*
19. *resistere alle pressioni dei compagni a fare cose che ti mettono nei guai?*
20. *Quando ti senti annoiato o scocciato, quanto sei capace di resistere alla tentazione di non andare a scuola?*
21. *Se i tuoi amici ti spingono a fumare, quanto sei capace di resistere?*
22. *Se i tuoi amici ti spingono a bere la birra, vino o liquori, quanto sei capace di resistere?*
23. *soddisfare i desideri dei tuoi genitori su quello che si aspettano da te?*
24. *soddisfare le richieste dei tuoi insegnanti?*
25. *realizzare quello che tu ti aspetti da te?*
26. *fare amicizia con le ragazze?*
27. *fare amicizia con i ragazzi?*
28. *Se sei insieme ai tuoi amici e state discutendo su qualcosa, quanto ti senti capace di parlare e dire la tua (opinione)?*
29. *lavorare in un gruppo?*
30. *dire quello che pensi, anche quando i tuoi compagni non sono d’accordo con te?*
31. *difendere i tuoi diritti quando vieni trattato ingiustamente?*
32. *Se qualcuno ti dà fastidio o ti prende in giro, quanto sei capace di cavartela?*
33. *Se qualcuno ti chiede di fare qualcosa di assurdo o di vietato, quanto sei capace di non farlo?*

**CPSE English Version**

“Translation modified from [Pastorelli, C., Caprara, G. V., Barbaranelli, C., Rola, J., Rozsa, S., & Bandura, A. (2001)] under a CC BY license, with permission from [Prof. Concetta Pastorelli], original copyright [2001].

*How well can you:*

1. *learn general mathematics?*
2. *learn Englih (literature, Grammar)?*
3. *learn history?*
4. *learn science?*
5. *finish homework assignments by deadlines?*
6. *study when there are other interesting things to do?*
7. *concentrate on school subjects?*
8. *take class notes of class instruction?*
9. *use the library to get information for class assignments?*
10. *organize your school work?*
11. *plan your school work?*
12. *remember information presented in class and textbooks?*
13. *arrange a place to study without distractions?*
14. *motivate yourself to do school work?*
15. *participate to class discussions?*
16. *learn sport skills?*
17. *learn regular physical education activities?*
18. *learn the skills needed for team sports (for example, basketball, volleyball, swimming, football, soccer)?*
19. *resist peer pressure to do things in school that can get you into trouble?*
20. *stop yourself from skipping school when you feel bored or upset?*
21. *resist peer pressure to smoke cigarettes?*
22. *resist peer pressure to drink beer, wine or liquor?*
23. *live up to what your parents expect of you?*
24. *live up to what your teachers expect of you?*
25. *live up to what you expect of yourself?*
26. *make and keep female friends?*
27. *make and keep male friends?*
28. *carry on conversations with others?*
29. *work in a group?*
30. *express your opinions when other classmates disagree with you?*
31. *stand up for yourself when you feel you are being treated unfairly?*
32. *deal with situations where others are annoying you or hurting your feelings?*
33. *stand firm to someone who is asking to do something unreasonable or inconvenient?*
